# Philadelphia Hospital

**Published:** 1838-01-03

**Authors:** 


### CLINICAL LECTURES.

### *PHILADELPHIA HOSPITAL.*

This institution is attached to the Philadelphia Almshouse, situated in the district of Blockley, on the western bank of the Schuylkill. Clinical lectures are here delivered, during the winter season, on Saturdays, from 10 o'clock, A. M. to half-past twelve, P. M., by the surgeons and physicians of the hospital, on duty at that time. Dr. Gibson lectures on Surgery during the first three months of the winter, and Dr. Harlan, during the remainder of the term. The medical lectures are given by Drs. Jackson and Gerhard.

*Saturday, 16th December.* Dr. Gibson introduced into the amphitheatre, a negro, with a tumor involving the upper jaw. This patient had been before the class on a previous occasion. An operation for the removal of the tumor having been determined on, Dr. Gibson announced his intention of performing it to-day: present Drs. Harlan, Horner, and Pancoast.

Upon a close examination of this case, said Dr. G., I find the tumor to be mixed up with the bones of the palate, and possibly extending to the antrum. Its origin is uncertain. It may arise from an epulis growing out so as to involve the upper jaw, or from a small tumor proceeding from the lining membrane of the antrum, or from caries of the bone or teeth, or it may be syphilitic or scrofulous. The patient has had it for two years. Possibly the upper jaw may be loosened, or it may still preserve its adhesions. The palate is involved, as far as the Eustachian tube, and likewise, the posterior nares. It is common for such tumors to arise chiefly from the gums. Mr. John Bell mentions them occurring from such origins, or from the glands in the parts. Sometimes the tumor forces itself so far into the bony structure, that it is impossible to get it out. It is necessary to operate as early as possible, whether the tumor be cancerous, sarcomatous, or arises from caries. In this case, it is my opinion, and that of the majority of my colleagues, that the man's only chance is an operation, to extirpate the disease. I cannot answer for success, although I have succeeded in worse cases. No surgeon can be responsible for results; I have known such trivial operations as the cutting out of a corn, to prove fatal. Where there is every prospect of a disease proving fatal, we are justified in attempting its removal by an operation. No one can predict the success, or ill success, that may ensue. I may instance, as a case in point, the perils of child-birth. Some women will suddenly expire from hœmorrhage, after a simple and speedy delivery; while others will have the parts torn to pieces with the forceps, or the perinæum lacerated, and yet no bad consequences result. This case requires an immediate and decided operation. The obstacles I must expect to encounter in performing it, are, hœmorrhage, and the difficulty and severity of the operation itself. I am not in the habit of attempting desperate operations. I have never attempted to take out the parotid gland, because I know it cannot be all taken out. I never operate where there is no chance of success; but, when there is a chance, if the patient wish to avail himself of it, my agency shall not be wanting. My method of performing this operation differs from that of the generality of surgeons. I shall not go to piddling about it; I shall not pull one tooth after another, and grope my way along slowly; but shall go to work with the chisel and hammer, and expedite the operation. One or two blows with the chisel and mallet will save the patient ten times the pain he would otherwise suffer.

[Dr. Gibson then proceeded to perform the operation. He removed both the upper maxillary bones, leaving only enough to support the bones of the nose. He accomplished the excision with knives, made for the purpose, the steel of which was remarkably well tempered, and the gouge and mallet. The actual cautery was used to arrest hœmorrhage. The operation occupied about twelve minutes. The result will be given hereafter.]

Dr. Gibson resumed: The only danger which I

apprehend, in this case, gentlemen, is the return of the disease. I cannot find any traces of the tumor, in the antrum; but if it have extended far back, it will be apt to grow again. If this occur, the application of the actual cautery will be necessary to keep down any fungus growth. I have removed, with the knife, all that is practicable. The tumor I discover to be osteo-sarcomatous. In all cases where I have operated for the removal of such tumors, I have invariably found them to return. I believe this patient will not get well eventually, but I have given him his only chance. If I had been in his place, I would have submitted to such an operation as I have performed on him. It is my rule never to undertake an operation which I would not be desirous to undergo myself, under similar circumstances.

Dr. Gibson terminated the lecture by presenting a specimen of morbid anatomy, illustrative of a case before the class on some antecedent occasion. We have been promised, but have not yet obtained the details of this case, which we reserve for our next.

Dr. Gerhard followed Dr. Gibson. He remarked, that he would to day call the attention of the class to certain acute febrile diseases. He presented to them a patient, admitted last evening into the wards under his charge, exhibiting a milder form of petechial typhus fever. The patient was a labouring man, a blacksmith by trade, who had been living in a house, in which there was no sickness. He had not therefore contracted the disease from the influence of contagion. He was taken sick on Tuesday week; felt weakness, giddiness and pain of the head; thinks he had a chill, but his recollection is very confused. He had diarrhœa: this however is an accidental symptom. He suffered from dizziness; his sleep was disturbed by dreams. He had no cough, which is unusual in the disease, particularly at this season of the year. You naturally ask the question, gentlemen, why I determine this to be a case of typhus fever. What are the diagnostic characters of the disease? and in what points does it differ from typhoid fever? from the case of the young woman, whom I showed you at the last lecture? The patient before us offers a very mild type of the disease; it is a case of typhus mitior. Hence, if you are enabled to draw the distinguishing line in this instance, you will do so the more easily, when the symptoms are graver and more decidedly marked.

Typhus fever is complicated with great alterations of the blood; hence we have petechiœ, hœmorrhages, and such other symptoms. I may here notice that the observations upon which these remarks are based, have been made in the wards of this hospital, where the disease has prevailed epidemically, with more or less violence, since the early part of 1836. These observations, published by me in the American Journal of the medical sciences, have been admitted by English and Irish physicians, particularly by Dr. Graves, as accurately descriptive of the disease, as it occurs in England and Ireland. It prevailed formerly in the wards of this hospital, when situated within the limits of the city; and, since its removal to this side of the river, in 1834, it has re-appeared, at the period I mentioned.

The first symptom to which I shall call your attention, is the eruption. It is, as you see, universal. It is not confined to a few rose-coloured spots, as in typhoid fever; but is rather like the eruption of measles. It is most thick upon the trunk, pervading to a less extent the face and neck, and still less, the extremities. There is also on the body of this patient an eruption resembling urticaria, which has appeared since last night. This is accidental, and not at all characteristic of the disease. The petechial spots were present, when the patient entered the house. He has no sudamina, although they are often to be found. I wish you to observe the appearance of stupor which he exhibits; the livid flush of his face, the suffusion of his eyes. His tongue is covered with a brown and fuliginous coating, and his teeth encrusted with a secretion of the same description. His pulse is soft, and yields under a slight pressure. It is 120 per minute. There is but little subsultus tendinum, as it is, at this period, as I told you, a mild case. The pathognomic signs are the stupor, the petechiæ, and the suffusion of the eyes. The disease may be recognised, although bronchitis, which is a very frequent symptom is absent.

The disease is, at this time, epidemic. This patient came from near the Schuylkill. There were no cases of the disease in the house with him, or in the neighborhood. It did not therefore arise from the influence of contagion. The disease is by no means necessarily contagious, but if you inhale the miasm arising from the patitent's body, in a crowded, ill ventilated apartment, you will be very apt to contract the disease. Thus the typhus occurring in camps, is almost always contagious; while in private practice it is rarely so.

The treatment in this case has been simple. It is better not to interfere with the natural progress of the disease. The only symptom, at present requiring attention, is the diarrhœa. I have therefore merely directed the patient's body to be freely sponged with milk-warm water; effervescing draughts to be exhibited, and shall treat the diarrhœa with small opiate injections, if the flaxseed enemata which I have ordered, shall fail in checking it.

The next patient I present to you offers an example of the same disease, but of a more severe grade. It is besides at a less advanced stage than the other, having existed only for six days. This patient came from a section of the city near the Delaware. Of course, then, his sickness is independent of that of the man just presented to you, who resided in the neighborhood of the Schuylkill. He was taken with dizziness, and with severe pains in his head, back, and limbs, and with a chill. For the last two days, his recollection has been confused, and we have been able to learn little more of his symptoms from himself. The last circumstance impressed on his memory, before its faculties were prostrated, was the chill; up to that point therefore, he can describe his symptoms accurately, and here, he fails. Though his memory is confused, he is quite sane. He has great disposition to somnolency. Remark his dull stupid look, and the listless manner in which his face is directed to one side. As the case is less advanced than the former, the flush is less livid, less dark, more bright, and more arterial. The eruption begins to shew itself, as he has reached the conclusion of the first week of the disease. Before, it appears, the disease is not recognized, unless it is known to be epidemic. The same remark applies to measles, smallpox and the exanthematous affections generally. You cannot diagnosticate a case of measles from the simple existence of coryza or catarrh. But if you couple these symptoms with the fact of its pervading the city epidemically, you may pretty safely make such a conclusion. If typhus fever be epidemic, you may consider the premonitory signs, as characteristic from the first. In this man, the eruption is just coming out. It resembles flea bites. The surface of his body, looks as if it had been pricked all over with a pin or needle, or as if insects had been biting it. The eruption is less distinct on his neck and limbs, than on his trunk. The suffusion of his eyes is well marked, arising from imperfect circulation in the conjunctiva. He has no delirium. He is agitated and restless; and the faculties of his mind are impaired owing to the loss of memory.— In this early stage, his feelings are very much such as result from intoxication, produced by a moderate quantity of alcoholic liquor. A remark commonly made by patients at the commencement of typhus fever, in describing their feelings is, that they feel as if they were a little tipsy. This patient has had no diarrhœa, no vomiting, and but little cough. His tongue is of a rosy colour; the papillœ are slightly elevated.

The young girl laboring under Typhoid fever, who was brought before the class, last Saturday, is to-day too unwell to appear. She is in an extremely critical state. She sleeps very badly, and has no appetite; has some subsultus, and labours under great stupor of mind. She is by no means convalescent, or even safe. I always look upon a patient as in great danger, till convalescence is well established. Until the patient's strength be well restored, he may at any time slip through your hands.

The next patient I show you, is an interesting case. He came into the house in such a situation as to be unable to give any account of himself. He was in a state of muttering delirium, could not sleep, and to such a degree was his mind affected, that he would cry out from time to time. He had a slight cough, with a mucous rhonchus on both sides of the chest. I ordered a blister to be applied to the back of his neck, and directed a small quantity of blood to be taken by cups from the same quarter. I considered it a case of slight meningitis affecting the base of the brain, and extending somewhat over the forepart of that organ. I gave the patient mercurials in small doses, with the object of slightly affecting his mouth. As soon as a moderate degree of ptyalism occurred, the intellectual faculties returned, and convalescence became pretty well marked. I consider this case worth noting, because it shows the good effects of small doses of mercurials, in sub-acute cerebral inflammations.

The next patient is a man about 40 years of age, who has been a drunkard, for a number of years. He came in, complaining of nausea, and great aversion to food, symptoms denoting irritation of the stomach, and extreme feebleness. Tonics were administered without affording relief. Afterwards, his head became affected. Then jaundice appeared, and the other symptoms were relieved, showing disease of the liver, dependent on an affection of the stomach, usual in persons indulging to excess in alcoholic liquors. In the beginning of jaundice, and indeed throughout its course, the cerebral symptoms which complicate it, differ from those denoting inflammation of the brain. There is no violent delirium: the face is of a pale, yellow hue. These patients cannot sleep; they usually labor under muttering delirium, and their countenances assume a wild look. What is the cause of these cerebral symptoms? It is an altered state of the blood, produced by the bile circulating in it, and in part by the deficiency of red globules. The affection of the brain, I look upon as the most important feature of jaundice. Unless this complication exist, there is no danger. You need pay little attention to any thing else, but if the brain be affected, deem your case a grave one. The proper treatment is, blisters, sinapisms, and other revulsives, to the back of the neck.

General bloodletting, to any extent, and even cups or leeches, are often mischievous. It is unsafe to bleed, when the state of the brain is dependent on the poison of bile in the blood. This state is a mere symptom, not a disease in itself. Many diseases, considered as such, are mere symptoms, such as the various kinds of dropsy. Here the effusion is a mere secondary effect. So the effusion of bile, and the cerebral affection in jaundice, are mere symptoms. The same remarks as to the impropriety of depletion, apply to cases of anemia, which affection is generally connected with jaundice. In all these cases, there is great tendency to sudden prostration, and you must avoid encouraging this tendency by large bleedings. There was a patient in my wards, about two years since, who was affected in this way. He had been a long time in the East Indies; and had the pale, yellow skin, indicative of anemia and bilious disorder. He was one day walking about the ward, when he was suddenly taken with difficult and laborious breathing. A state of coma ensued, which in a few hours ended in death; and no lesion of the brain was afterwards discoverable. In this case, you have a striking instance of the impropriety of bleeding in anemia, or in jaundice, in which there is more or less anemia. I call your attention to the alteration of skin apparent in this patient. You observe the yellow tinge which pervades it. The patient manifests great weakness. His stomach is uneasy. His appetite is poor. His tongue, you see, is white, swollen, with indentures in the corners. His pupils are contracted, which is unusual, and is owing to a previous attack of mania a potu.

A day or two since, a black man, affected with a chronic tubercular disease, having left the wards and been exposed to the cold, was taken with influenza. A sudden congestion followed, and he died from effusion into the bronchial tubes, and the substance of the lungs. Effusion into the lungs occurs from time to time in the winter season. The patient now introduced has been affected with dropsy of the lower extremities. A day or two since, he was suddenly taken with oppression of the chest. There was a sub-crepitant rhonchus, wheezing and rattling respiration. It was a case of effusion of thin serum into the bronchial tubes and substance of the lungs. The carbonate of ammonia was freely exhibited; blisters were applied to the chest, and sinapisms to the extremities. These remedies produced relief. Such cases require immediate attention, otherwise the patient perishes. We lose many cases in the wards of this hospital. especially amongst patients of bad constitutions. The treatment which I recommend is, the internal use of the diffusible stimulants, such as the carbonate of ammonia, Hoffman's anodyne, sulphuric ether, hot tea, &c. With these, combine revulsives to the extremities and chest. The existence of dropsical effusions predisposes to effusions into the bronchial tubes, and was the case with the patient before you. He is also in a state of dementia. This class of persons are particularly liable to these thoracic diseases, and, what is singular, they often complain of pain not in the part affected, but in some distant organ, as you may remark is the case with this individual, who speaks only of pain in his knees.

The disappearance of œdema usually coincides with effusion into the chest. Hence, the sudden removal of dropsical effusion is to be dreaded. I recollect a case of dropsy which was attended by two respectable physicians of this city. The effusion not disappearing under their hands, they were discharged, and a homœopathic doctor employed. From some cause or other, sudden diuresis was brought about. The patient was pronounced well, but in the course of two or three days he suddenly fell from his chair, and died. I remember also a case occurring in this house, in which death ensued twenty-four hours after a dropsy was suddenly removed. In the case before you, nature has suddenly cured the dropsy by throwing the effusion into the chest; which transference has probably been brought about by the sudden change in the weather. The symptoms have abated since the application of blisters, and the administration of carbonate of ammonia. I told you that lunatics and paralytics are particularly subject to this sort of affection. I may remark, that they are rarely attacked by acute pneumonia. They are also specially liable to some other diseases, as for example, typhus fever; while, on the other hand, pthisical patients rarely take it, when it is epidemic here.

I shall conclude the lecture with one other case, illustrating the subject of paralysis, and diseases of the brain, which I intend to take up and discuss at length, on a future occasion. I wish to call your attention to-day to a single case, in an advanced stage of the disease, to make the subject somewhat familiar to you, before I come to discuss it at large. This patient presents a form of paralysis, which you will not often meet with. It occurs in individuals who are insane, or whose minds are enfeebled. It is important in a pathological, but not in a therapeutic point of view. All affections of this sort are utterly intractable. This man entered the house, about two months since. His symptoms at that time, were a slight staggering in walking, and thickness of speech. There was at first no positive loss of motion. His arms were a little affected. There was no distortion of countenance, but merely an expression of dull stupor, with a look of anxiety. He faltered in his speech, and displayed signs of feebleness and imbecility of intellect. When this disease occurs in lunatics, violent insanity often supervenes; and strong incoherence of ideas may take the place of monomania. This patient, as you observe, cannot now walk at all. The functions of his bladder are impaired. His urine is drawn off by a catheter; he has lost control over the muscular coat. His hands and arms are equally rigid. The fingers, you see, are in a half flexed position, not entirely closed; his grasp is feeble. These symptoms, constantly increasing, denote paralysis of the insane. They are occasioned by an alteration of the cortical substance, and effusion between the membranes of the brain. There is no blood thrown out. This disease is invariably fatal.

At this point, Dr. Gerhard terminated his remarks.

*Saturday, 23d December.* Owing to the absence of Dr. Gibson, there was no clinical lecture on surgery. At a quarter past eleven, Dr. Gerhard appeared. The attention of the class was called, by Dr. Jackson, who was present, to the epidemic character which typhus fever is now assuming in this city. Two cases had occurred within the last two weeks, in his private practice, the first he had ever met with. He considered the disease to be gradually spreading, and would not be surprised to see it, before long, prevailing to an alarming extent. All the acute cases in the hospital are, at this time, taking on a low type.

Dr. Gerhard brought before the class the patient whom he had exhibited last Saturday, as a mild case of typhus fever, in the height of the disease. This man, said Dr. G., is now convalescent. There is still to be noticed a slow degree of action in his capillaries, with a moderate heat of skin. I have treated him principally with the neutral mixture, together with spongings with a solution of chloride of soda, and mild stimulating sudorifics.

I present to you the young girl whom I mentioned as too ill to appear before you last Saturday. She is now quite well, with the exception of a slough on the sacrum. This case has been one of much interest, and I propose noticing it with some detail. She came into my ward on the 29th of last November, having been ill for about 10 days before, during which time she suffered from great prostration, attended with loss of appetite and diarrhœa. Her symptoms, at the time of her admission into the hospital, were stupor, constant disposition to somnolency, flush of the face, dry tongue, tympanitis, and a diarrhœa of seven bloodless discharges, during the twenty-four hours. Pulse, soft and feeble, 104 per minute. She had a loose cough, with a sibilant rhonchus. There were no sudamina, but some rose-colored spots. These symptoms became aggravated, accompanied by delirium, restlessness, and jactitation, with subsultus tendinum, forming a well marked case of typhoid fever. I prescribed small doses of calomel, ipecacuanha, and opium. The febrile symptoms abated somewhat, but the degree of prostration became alarming; I ordered wine, tonics, and nourishing food, to meet these symptoms. This plan of treatment is proper, if the exhaustion be dangerous, otherwise I think it is better to abstain from it. Revulsives to the shoulders and extremities were also, from time to time, directed, with injections of lac assafœdita and camphor. I succeeded in checking the diarrhœa with small opiate injections. In typhoid fever, ten drops of laudanum is enough for a dose; you cannot give more without danger to the cerebral symptoms, and a moderate dose answers every purpose. Never begin with more than twenty drops, and very rarely go beyond forty, provided always the enema be retained. This girl has been, throughout the affection, in a most critical state, from her great feebleness and prostration. I have been treating her for some time past with tonics, and can to-day pronounce her well, with the exception of the slough I spoke of. This is doing well under the application of emollient poultices and simple dressings.

I next present to you, gentlemen, some specimens of morbid anatomy, to which I request your particular attention. The examination of this brain which I show you, involves questions not only of pathology, but of medical jurisprudence. It is the brain of a drunkard, who was admitted into the hospital as a surgical patient, for an injury to the head from a fall. He died, presenting symptoms both of delirium tremens and of inflammation of the brain. It becomes a question, gentlemen, and such a question as you will probably be hereafter called upon to answer in a court of justice, what was the cause of this man's death. Did he die from the injury he had received, or from the effects of excessive drinking? His death took place within three or four days after his admission, while the inflammatory symptoms were at their height, and before the occurrence of collapse. The brain, as you see, exhibits decided marks of inflammation of its membranes. The man died of meningitis. This I determine, because the lesions indicating it are better marked than those which are characteristic of mania a potu. In the latter we find paleness of the brain, and effusion into the ventricles. In this case you notice the reverse. The exterior of the brain is of a bright hue, the small vessels remarkably injected. This is best seen between the hemispheres of the cerebrum and between the great fissure. Upon a still further examination, you perceive that the surface of the arachnoid is of a yellow opaline tint, adhering very closely to the cortical substance, which is of a pretty firm consistence. There are slight symptoms of inflammation in the interior of the brain.

Here is another brain, of a man who had an attack of apoplexy, about two years since. If you examine the brain, immediately after death from apoplexy, you find usually, as most of you know, a clot of blood in the corpus striatum, or in the thalamus. If the patient live, the liquid parts of this clot are absorbed, and a cyst remains, or a mere mass of cellular substance, as in this instance. Hence, you see the impossibility of a perfect cure of a paralysis of the opposite side, from the constant existence of this obstruction. You observe the shrunken condition of the corpus striatum on this side, although a cicatrix has been formed. A restoration of perfect motion to the opposite side is impossible, if the effusion of blood have been large. If it be small, the fibres of the brain may not be ruptured, and a cure may take place.

This liver, on the table, is that of a black man, who was admitted into the hospital last spring, with pleurisy of the right side. His convalescence was slow, which fact, in connection with some other symptoms, made me suspect the existence of tubercular disease, although there were no local signs whatever. He was discharged, but, towards autumn, returned with an enlargement of the left hypochondriac region, extending into the epigastrium, with severe lancinating pain, arising apparently from peritonitis. Gradual emaciation took place, and the patient died on the 18th December. Upon examination after death, I found a few very small tubercles in the lungs, and strong adhesions throughout the pleuræ. The liver, especially the left lobe, is, you see, enormously enlarged, and would extend beyond the umbilicus. In the right lobe are several tubercular masses, half softened, the largest of which is the size of a pigeon's egg. It is the largest tubercle I ever saw in the liver. Near these you see this abscess, containing about two ounces of well formed, laudable pus. There were adhesions between the liver and ribs opposite to these morbid collections. There were tubercles in the lymphatic glands; the gland near the pancreas was composed almost entirely of tubercles, which were also found in the bronchial glands, but not in the cervical. It was a case of internal scrofula. Tubercles in the liver are excessively rare; carcinoma is a frequent occurrence. The latter is apt to be mistaken for the former. I hold upon my knife a small slice of the liver, to show the fatty condition of its substance: this condition is an ordinary complication of phthisis. This case, gentlemen, exemplifies an important pathological law: the occurrence of two consecutive serous inflammations, in young subjects, and uncombined with acute rheumatism, demonstrates the existence of tubercles. We have made, then, today, two deductions of much interest, from the study of pathological anatomy—that just mentioned, and the impossibility of curing the paralysis dependent on apoplexy, when the effusion of blood upon the brain has been considerable.

Dr. Gerhard announced that Dr. Jackson would, at the next lecture, take up the subject of diseases of the heart, and of neuralgia.

